# Relation between Mechanical Hardening and Nitrogen Profile of PBII Nitrided Titanium Alloy

**DOI:** 10.3390/ma15249028

**Published:** 2022-12-17

**Authors:** Valérie Parry, Eric Le Bourhis, Luc Pichon, Michel Drouet

**Affiliations:** 1 SIMaP, Université Grenoble Alpes, CNRS, Grenoble INP, F-38000 Grenoble, France; 2 Institut P’, CNRS, Université de Poitiers, Bd Marie & Pierre Curie-TSA 41123, F-86073 Poitiers, France

**Keywords:** titanium alloy, nitridation, plasma, implantation, structure change, mechanical performance

## Abstract

Surface treatments of Ti-6Al-4V alloys are of utmost importance for biomedical applications since they allow for tribological gain. Here, Ti-6Al-4V disks have been PBII nitrided at either 500, 600, 700 and 800 °C. A set of techniques (XRD, SEM-EDS, EBSD and GDOES) was used to characterize the surface microstructural and chemical changes. Nanoindentation was used to assess the induced changes in terms of mechanical properties. Two types of nitrided domains are revealed. Starting from the surface, a nitride bilayer composed of δ-TiN/ϵ-Ti2N with enhanced surface resistance is supported by an α-Ti(N) solid solution formed at depth. Hardness values peak at 12–14 GPa at the surface, which is almost twice as large as the bulk value (about 7 GPa). For the moderate temperatures used here, a deep (10–15 µm) and strong hardness (14 GPa) enhancement together with a smooth gradient can be achieved.

## 1. Introduction

The ageing of our populations as well as recurring diseases and trauma have stimulated a high demand for bone reparations. The development of prostheses is challenging as the materials and design used should be adapted to bone functions in terms of mechanical properties, while cytocompatibility, osseointegration and inertness in the long term remain of utmost importance. Titanium alloys (notably Ti-6Al-4V) present some advantages, showing lesser elastic strength than steel, and therefore are used for implants [1]. Their surface has to be carefully assessed, since wear and chemical leaching have to be prevented. Bulk titanium alloys show poor tribological properties unless their surface is treated. One alternative is to deposit a hard coating on top of the solicited parts. A second alternative comprises the nitriding of the protheses so as to harden its subsurface and generate a mechanical gradient. However, nitriding of titanium alloys at moderate temperatures remains very challenging. The objective is to harden the surface while retaining chemical stability as well as preserving biocompatibility [2,3], without affecting the mechanical properties of the core (e.g., fatigue strength [4,5]). In the case of treatments above 700–800 °C, thermal nitriding leads to nearly a stoichiometric nitride layer (<1 μm) and a diffusion layer by inserting nitrogen into the crystal structure [6,7]. Although the hardness and the resistance to frictional wear can be improved, the fatigue behavior may be affected. In the worst case, fracture occurs earlier because of the aging of the bulk material. At lower temperatures of 400–500 °C, a nitrogen solid solution in α-Ti or the intermediary compound ϵ-Ti_2_N may be obtained [8,9,10]. So far, the gain in wear resistance remains insufficient, as the modified layer is very limited. Therefore, ion implantation has been proposed to produce TiN nitride while retaining core characteristics [11]. This process step has been implemented in the production lines for hip prostheses with in-line implanter systems [12,13,14], although the additional costs remain detrimental. In fact, the available ion current is low and several manipulations are needed to treat pieces of complex shape. In contrast, a plasma can embed three-dimensional pieces. Hence, plasma-based ion implantation (PBII) combines the advantages of both implantation and plasma treatments and was first successfully used to treat steel [15,16] and then titanium. The treatment of titanium and Ti-6Al-4V has been reported to allow for performance to be tailored, namely, corrosion [17,18], hardness [19,20] and wear [8,21,22,23]. In previous works, commercially pure titanium was chosen to develop fundamental studies on the nitriding treatment influence on the mechanical properties as this avoids the complexity resulting from the dual α/β microstructure [24,25]. In the present paper, experiments have been conducted on Ti-6Al-4V alloy, which accounts for 50% of the titanium alloys market. We used PBII treatments at temperatures that allow the bulk performance to be retained while hardening the surface. The treatment temperature domain has been explored so as to determine the best compromise between the temperature and the time of treatment. Instrumented indentation has been used to monitor the in-depth changes of the mechanical properties [26,27]. To do so, it has been necessary to circumvent the roughness generation that is known to dramatically affect the results [16,28,29].

## 2. Experimental Techniques

### 2.1. Material and Nitriding

First, 10 mm diameter Ti-6Al-4V disks were mechanically ground with abrasive SiC papers and then polished with diamond spray (1 μm). Samples were then ultrasonically degreased in acetone and in ethanol and dried with nitrogen gas before the nitriding process. Ti-6Al-4V composition supplied by Goodfellow is displayed in Table 1.

Ti-6Al-4V specimens were treated in a homemade thermally assisted plasma-based ion implantation (PBII) set-up [30]. Nitriding was carried out at four different temperatures, 500, 600, 700 and 800 °C, in a mixture of 90% N_2_–10% H_2_ at a pressure of 1 Pa. The implantation parameters used are as follows: a constant time of 240 min, an acceleration voltage of 25 kV, a repetitive rate of 200 Hz and a 10 μs pulse length leading to a total effective implantation time of 29 s.

### 2.2. Structural Characterizations

X-ray diffraction (XRD) measurements were performed with monochromatic CuKα radiation (Siemens diffractometer D5005) with a grazing incidence of 5°, which corresponds to a probing depth of less than 2 μm. Cross sections were prepared according to the polishing procedure used for cross-sectional nanoindentation (see Section 2.3) described in [24]. Chemical and microstructural observations were performed on cross sections using a SEM Zeiss Ultra 55 field emission gun (FEG) equipped with a SSD Bruker X-ray detector for qualitative element depth profiling and a SEM Geminy equipped with electron back scattered diffraction (EBSD, TSL-EDAX) under a voltage of 15 kV and a current of 10 nA. Image binning was set to 2 × 2. Qualitative nitrogen, titanium, aluminium and vanadium depth profiles were also obtained via glow discharge optical emission spectroscopy (GDOES) and carried out on a Jobin-Yvon GD profiler. Conditions of sputtering and acquisition were strictly identical for all samples. After the analysis, the crater depths were measured to determine the average sputtering rate. No quantitative calibration of the different element concentrations could be performed, so the extracted profiles remain qualitative.

### 2.3. Mechanical Characterization

Cross sections were prepared for nanoindentation testing. This is a difficult procedure in the presence of a mechanical gradient [24]. Briefly, special care was taken to ensure both the surface flatness and the absence of work hardening. A dedicated titanium sample holder was designed to grab two sections of the treated specimens. The holder was then used to carefully polish the specimen surfaces to colloidal silica. Then, the specimens were deformed using a Berkovich indenter with a NHT instrument from Anton Paar [31]. Hardness profiles were determined probing the cross sections at increasing distance from the treated surface, using a distance between indents large enough to rule out interference.

## 3. Results and Discussion

### 3.1. Structural Characterizations

**Figure 1:**
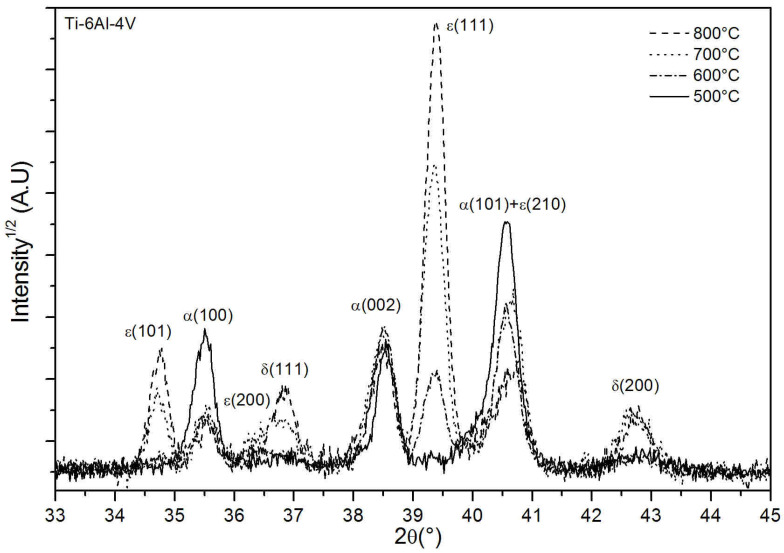
X-ray diffraction results with incidence angle of 5° performed on Ti-6Al-4V specimens treated with PBII (25 kV, 200 Hz, 10 μs) for 240 min at either 500, 600, 700 or 800 °C in a mixture of 90% N_2_–10% H_2_ at 1 Pa.

X-ray diffraction with an incidence angle of 5° was performed on Ti-6Al-4V specimens treated for 240 min at 500, 600, 700 and 800 °C. Results are shown in Figure 1. The diffraction peaks are identified as the hexagonal α-Ti (JCPDS 00-044-1294) and as two phases of titanium nitride, namely, ϵ-Ti2N (JCPDS 04-004-3072) and δ-TiN (JCPDS 00-038-1420). A (110) β-Ti phase peak, observed at 39.2° on the as-received sample (not shown here), is not detected anymore after the nitriding process. For the treatment performed at 500 °C, ϵ-Ti_2_N is not observed, only the most intense (200) peak of δ-TiN is detected with a very low intensity because of the low diffracting volume. In fact, this layer cannot be detected on SEM cross-sectional images, as discussed in more details below. With increasing treatment temperature, the nitride phases peaks grow, especially ϵ(111), while the intensities of the (100) and (101) α-Ti peaks decrease. The increase of peak intensity of TiN and Ti_2_N indicates larger diffracting volumes, presumably because of a thicker layer being formed, although it is difficult to be quantitative. Noticeably, the (002) α-Ti peak intensity remains constant. This modification of the relative intensity of α-Ti peaks could be the consequence of some degree of texturation of the nitrogen-induced recrystallized α-Ti layer formed underneath the nitride layers. In fact, this recrystallized layer becomes thicker with increasing treatment temperature. A slight line shift towards the small angles of the (002) α-Ti peak is observed. It can be linked to two phenomena that coexist: a dilatation of the α-Ti cell due to nitrogen incorporation and the presence of compressive residual stresses induced by the presence of interstitial nitrogen. As expected from the preferential expansion of the α-Ti cell along the c axis upon nitrogen uptake [32], almost no low angle shift is detected for the (100) peak.

**Figure 2:**
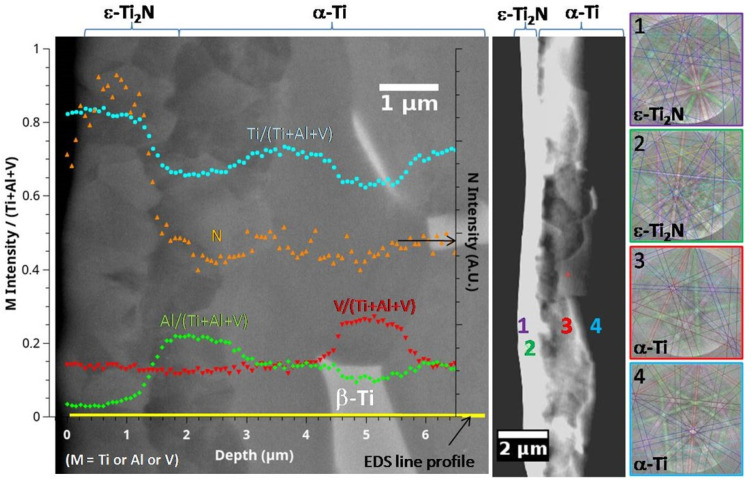
SEM, EDS and EBSD results obtained on cross section of Ti-6Al-4V sample treated via PBII (25 kV, 200 Hz, 10 μs) for 240 min at 800 °C in a mixture of 90% N_2_–10% H_2_ at 1 Pa.

**Figure 3:**
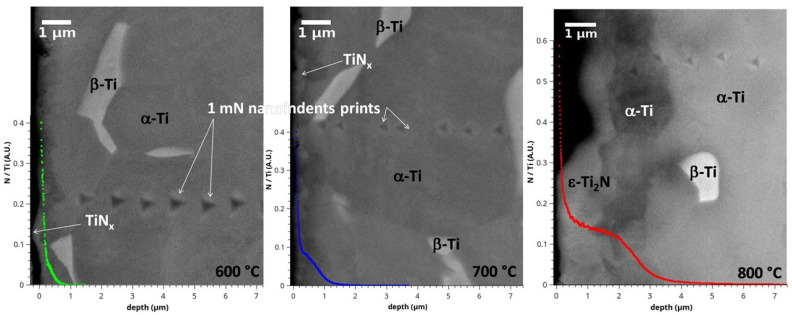
Cross-sectional SEM views after nanoindentation tests at 1 mN and N/Ti GDOES profiles of Ti-6Al-4V sample treated via PBII (25 kV, 200 Hz, 10 μs) for 240 min at either 600, 700 or 800 °C in a mixture of 90% N_2_–10% H_2_ at 1 Pa.

A cross-sectional SEM micrograph, EDS profile and EBSD phase identification performed on the sample nitrided at 800 °C are shown in Figure 2. PBII treatment performed at an elevated temperature induces important microstructural changes. According to Figure 2, the surface area is composed of two layers with rough interfaces of thickness slightly larger than 1 μm for the external one and about 1.5 μm for the internal one with a grain size of around 500 nm. These thicknesses are in good agreement with the qualitative GDOES profiles obtained on this sample (see Figure 3 right hand side). According to the EDS profile and EBSD analysis (shots 1 and 2, Figure 2), the top layer is mainly composed of ϵ-Ti_2_N. Surface nitriding leads to counterdiffusion of aluminium and its accumulation right below the nitride layer, as confirmed via the EDS profile (Figure 2). Recrystallization is observed in the aluminium-rich layer leading to a small grain microstructure. However, Al enrichment does not seem to lead to Ti_3_Al phase formation (EBSD shots 3 and 4, Figure 2), contrary to what is observed at 1100 °C [33] or when Ti-6Al-4V alloy was glow discharge nitrided at 680 °C/4 h and 750 °C/3 h [34]. Vanadium-rich β-Ti grains are observed a few microns below the two layers marking the end of the diffusion zone of alphagen nitrogen. In the most superficial region affected by the treatment N indeed inserts into β-Ti, and as it is a strong α stabilizer, changes it to α-Ti. As a consequence, almost no β-Ti grains remain. Hence, the nitrided layer formed in this study consists of δ-TiN and ϵ-Ti2N imbricated small crystals supported by an α-Ti(N) solid solution.

High temperature treatments induce a high value of surface roughness linked to the important degree of chemical modifications and also to the presence of a relatively thick nitride layer. It has to be noted that such a roughness value is not acceptable for nanoindentation experiments and explains the need of cross sections, as detailed above.

### 3.2. Mechanical Properties

**Figure 4:**
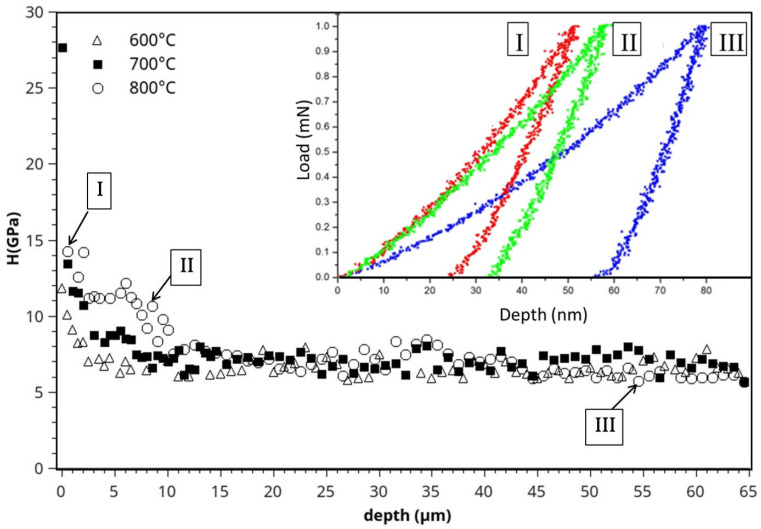
Hardness profiles for 3 treatments at 600 °C, 700 °C and 800 °C. Inset loading-unloading curves obtained for sample treated at 800 °C in three zones (I, II, III) defined on the profile.

Figure 3 presents an example of 1 mN nanoindentation print lines obtained on samples treated at three different temperatures. These correspond to cross-sectional SEM micrographs of the samples nitrided at either 600, 700 or 800 °C. In all three cases, several marks arranged in lines starting from the nitrided surface can be observed. Close to the nitrided side, the marks are revealed to be smaller in size, since the surface is hardened in contrast to the interior as a result of the nitriding process. The 1 mN loading and unloading curves corresponding to the treated and untreated parts of the sample nitrided at 800 °C are shown in Figure 4 inset. The curves differ dramatically since the indenter maximum depth in the nitride layer is about 60% of the bulk value and about 72% in the diffusion zone.

The evolutions of hardness are plotted as a function of the distance for three of the used temperatures in Figure 4. A strong evolution in the hardness values is observed at the vicinity of the treated surface, revealing the mechanical changes induced by the nitriding process regardless of the temperature used. The profiles can be divided into three domains (I, II, III in Figure 4). The first one is about 3 μm in depth and is characterized by a strong decrease in the mechanical properties. In the second domain, which extends from 3 to 10–15 μm in depth depending on the treatment temperature, the decrease is less abrupt. Only at 800 °C, do we observe a small plateau of about 5 μm depth. The plateau results from the formation of a Ti_2_N as already revealed with GDOES because of the N saturation of the alloy, as modeled in ref [1]. The third domain corresponds to the bulk.

Interestingly, the decrease in hardness is more gradual when compared to stainless steel nitrided via PBII, where an abrupt profile has been reported [16,35]. So far, hardening reaches a depth as large as 10–15 μm depending on the treatment temperature with values peaking at 14 GPa (resp. 12 GPa) at the surface after a treatment at 800 °C (resp. 600 °C), which is almost twice as large as the bulk value (about 7 GPa). Elastic behavior can also be extracted from instrumented indentation using the unloading curve [31]. The reduced modulus (Figure S1) was observed to decrease continuously from 150 GPa to about 130 GPa in the bulk. The bulk value is in relatively good agreement with literature (assuming a Poisson ratio of 0.3, E_Ti_ = 120 GPa [7,31]).

Young’s moduli of δ-TiN reported in the literature are set in a large range (210 GPa to 320 GPa) [26,36,37]. These values differ dramatically from those extracted here. The Young’s modulus of δ-TiN is reported to be affected by its stoichiometry, which is affected by the presence of vacancies in the nitrogen sublattice [38]. The results indicate that the nitrided layer obtained here is not stoichiometric. In fact, as discussed in Section 3.1, the superficial layer was revealed to be a mixture of δ-TiN and ϵ-Ti_2_N. It should be noted that when the grazing incidence is about 5°, the probe extends only about 2 μm. Hence, the layer analyzed in Figure 2 may also contain α-Ti. Finally, we assumed that the superficial layer comprises δ-TiN and ϵ-Ti_2_N imbricated small crystals supported by an α-Ti(N) solid solution. Such a complex substructure has to be at the origin of the discrepancy between the obtained results and the literature.

Tribological gain can be predicted from the hardness versus elastic modulus ratios [31]. Hardness variations dominate here over elastic modulus ones so that the performance is expected to be improved by a factor about 2.

## 4. Conclusions

The present results depict the preponderant role of temperature on both the structure and the mechanical behavior of the surface layers obtained during PBII surface treatments of Ti-6Al-4V titanium alloy. Whatever the studied temperature, the PBII nitriding process resulted in the formation of two nitride domains. The most superficial one is a “composite” organization of δ-TiN/ϵ-Ti_2_N, while at depth an α-Ti(N) solid solution is formed. At the lowest studied temperatures (500 °C–700 °C), the response of these two domains to a nanoindentation test presents a continuous evolution of the property gradient with a strong hardening of the surface. The in-depth α-Ti(N) domain presents an increase of hardness with nitrogen insertion into the α-Ti structure, while much stronger changes are observed at the extreme surface, where a δ-TiN/ϵ-Ti_2_N composite is formed. The α-Ti(N) is expected to prevent the hard and fragile nitride surface layer from breaking (the eggshell effect). At 800 °C, the diffusion process becomes more efficient and leads to a plateau of medium hardness and a smooth mechanical properties gradient in the diffusion layer. The mechanical properties extracted from the bulk of the sample remain equivalent to those of Ti-6Al-4V indicating the preservation of the core properties of the sample. This later aspect is of primary importance for practical applications in medical or aeronautic domains.

## Figures and Tables

**Table 1:** Chemical composition of Ti-6Al-4V [wt%].

Element	Ti	Al	V	O	Fe	C	N
Ti-6Al-4V	Bal.	5.5–6.75	3.5–4.5	0.2	0.4	0.1	0.05

## Data Availability

The data presented in this study are available on request from the corresponding author.

## Supplementary Materials

The following supporting information can be downloaded at: https://www.mdpi.com/article/10.3390/ma15249028/s1, Figure S1: Reduced elastic modulus profiles for 3 treatments at 600 °C, 700 °C and 800 °C.

## References

[1] Zhecheva A., Sha W., Malinov S., Long A. (2005). Enhancing the microstructure and properties of titanium alloys through nitriding and other surface engineering methods. Surf. Coat. Technol..

[2] da Silva J.S.P., Amico S.C., Rodrigues A.O.N., Barboza C.A.G., Alves C., Croci A.T. (2011). Osteoblastlike Cell Adhesion on Titanium Surfaces Modified by Plasma Nitriding. Int. J. Oral Maxillofac. Implant..

[3] Clem W.C., Konovalov V.V., Chowdhury S., Vohra Y.K., Catledge S.A., Bellis S.L. (2005). Mesenchymal stem cell adhesion and spreading on microwave plasma-nitrided titanium alloy. J. Biomed. Mater. Res. Part A.

[4] Oliveira V., Cioffi M., Barboza M., Landers R., Schmitt B., Tapia D., Voorwald H. (2018). Plasma immersion ion implantation (piii) influence on ti-6al- 4v alloy: Frequency effect. Int. J. Fatigue.

[5] Takesue S., Kikuchi S., Akebono H., Morita T., Komotori J. (2020). Characterization of surface layer formed by gas blow induction heating nitriding at different temperatures and its effect on the fatigue properties of titanium alloy. Results Mater..

[6] Fouquet V., Pichon L., Drouet M., Straboni A. (2004). Plasma assisted nitridation of Ti-6Al-4V. Appl. Surf. Sci..

[7] Hainsworth S., Soh W. (2003). The effect of the substrate on the mechanical properties of tin coatings. Surf. Coat. Technol..

[8] Lanzutti A., Raffaelli A., Magnan M., Fedrizzi L., Regis M., Marin E. (2019). Microstructural and mechanical study of an induction nitrided Ti gr.5 hip prosthesis component. Surf. Coat. Technol..

[9] Morita T., Takahashi H., Shimizu M., Kawasaki K. (1997). Factors controlling the fatigue strength of nitrided titanium. Fatigue Fract. Eng. Mater. Struct..

[10] Costescu C.I., Heuser B.J. (2000). The effect of a nitrogen-rich surface layer on the sub-surface deuterium (hydrogen) concentration distribution in titanium. Surf. Sci..

[11] Wilson A., Leyland A., Matthews A. (1999). A comparative study of the influence of plasma treatments, PVD coatings and ion implantation on the tribological performance of Ti-6Al-4V. Surf. Coat. Technol..

[12] Alipour R., Khani A.A., Mohammadi R., Rostami S. (2016). The effect of formation of titanium nitride thin film on surface characteristics of titanium by nitrogen ion implantation. J. Chem. Res..

[13] Yas’kiv O.I., Pohrelyuk I.M., Fedirko V.M., Bonchyk O.Y., Kravchyshyn T.M. (2007). Properties and structural-phase state of the surface layers of titanium after combined nitriding. Mater. Sci..

[14] Aghajani H., Motlagh M.S. (2017). Effect of temperature on surface characteristics of nitrogen ion implanted biocompatible titanium. J. Mater. Sci. Mater. Med..

[15] Collins G., Hutchings R., Short K., Tendys J. (1998). Ion-assisted surface modification by plasma immersion ion implantation. Surf. Coat. Technol..

[16] Marot L., Le Bourhis E., Straboni A. (2002). Improved nitridation efficiency and mechanical property of stainless steel surface after N-2-H-2 plasma nitridation at low temperature. Mater. Lett..

[17] Morita R., Azuma K., Inoue S., Miyano R., Takikawa H., Kobayashi A., Fujiwara E., Uchida H., Yatsuzuka M. (2001). Corrosion resistance of tin coatings produced by various dry processes. Surf. Coat. Technol..

[18] Lacoste A., Béchu S., Arnal Y., Pelletier J., Vallée C., Gouttebaron R., Stoquert J. (2002). Nitrogen profiles in materials implanted via plasma-based ion implantation. Surf. Coat. Technol..

[19] Berberich F., Matz W., Kreissig U., Richter E., Schell N., Moller W. (2001). Structural characterisation of hardening of Ti-Al-V alloys after nitridation by plasma immersion ion implantation. Appl. Surf. Sci..

[20] Geng Y., McCarthy E., Brabazon D., Harrison N. (2020). Ti6al4v functionally graded material via high power and high speed laser surface modification. Surf. Coat. Technol..

[21] Stratton P., Graf M. (2010). Wear of diffusion hardened Ti–6Al–4V sliding against tool steel. Wear.

[22] Johns S., Bell T., Samandi M., Collins G. (1996). Wear resistance of plasma immersion ion implanted Ti6Al4V. Surf. Coat. Technol..

[23] Alonso F., Rinner M., Loinaz A., O˜nate J., Ensinger W., Rauschenbach B. (1997). Characterization of ti-6a1-4v modified by nitrogen plasma immersion ion implantation. Surf. Coat. Technol..

[24] Fouquet V., Le Bourhis E., Pichon L., Drouet M., Straboni A. (2004). Elastic– plastic resistance profile of pbii nitrided titanium. Scr. Mater..

[25] Drouet M., Pichon L., Dubois J., Bourhis E.L., Christiansen T. (2020). Surface engineering of titanium by multi-interstitial diffusion using plasma processing. MATEC Web Conf..

[26] Barbieri F., Otani C., Lepienski C., Urruchi W., Maciel H., Petraconi G. (2002). Nanoindentation study of Ti6Al4V alloy nitrided by low intensity plasma jet process. Vacuum.

[27] Berberich F., Matz W., Richter E., Schell N., Kreisig U., Möller W. (2000). Structural mechanisms of the mechanical degradation of ti–al–v alloys: In situ study during annealing. Surf. Coat. Technol..

[28] Meletis E., Cooper C., Marchev K. (1999). The use of intensified plasma-assisted processing to enhance the surface properties of titanium. Surf. Coat. Technol..

[29] Kunert M., Baretzky B., Baker S., Mittemeijer E. (2001). Hardness-depth profiling on nanometer scale. Metall. Mater. Trans. A Phys. Metall. Mater. Sci..

[30] Marot L., Drouet M., Berneau F., Straboni A. (2002). High temperature plasma based ionic implantation of titanium alloys and silicon. Surf. Coat. Technol..

[31] Le Bourhis E. (2014). Glass Mechanics and Technology.

[32] Bars J., Etchessahar E., Debuigne J. (1977). Kinetics, diffusion and morphology of Titanium nitriding using high-temperature nitrogen—Mechanical and structural properties of solid Ti-alpha-nitrogen solutions. J. Less-Common Met..

[33] Lee H., Kang H., Kim J., Shin H.-K., Lee J., Huh S.-H., Sung J., Lee H.-J. (2014). Inward diffusion of al and ti3al compound formation in the ti–6al–4v alloy during high temperature gas nitriding. Surf. Coat. Technol..

[34] Morgiel J., Wierzcho’n T. (2014). New estimate of phase sequence in diffusive layer formed on plasma nitrided ti-6al-4v alloy. Surf. Coat. Technol..

[35] Tromas C., Stinville J.C., Templier C., Villechaise P. (2012). Hardness and elastic modulus gradients in plasma-nitrided 316L polycrystalline stainless steel investigated by nanoindentation tomography. Acta Mater..

[36] Patsalas P., Charitidis C., Logothetidis S. (2000). The effect of substrate temperature and biasing on the mechanical properties and structure of sputtered titanium nitride thin films. Surf. Coat. Technol..

[37] Musil J. (2000). Hard and superhard nanocomposite coatings. Surf. Coat. Technol..

[38] Guemmaz M., Mosser A., Ahuja R., Parlebas J. (2001). Theoretical and experimental investigations on elastic properties of substoichiometric titanium nitrides: Influence of lattice vacancies. Int. J. Inorg. Mater..

