# Supplementary material for: Relation between Mechanical Hardening and Nitrogen Profile of PBII Nitrided Titanium Alloy
Source: Materials (Basel). 2022 Dec 17;15(24):9028. doi: 10.3390/ma15249028 (PMC9785563; doi:10.3390/ma15249028)

Relation between Mechanical Hardening and Nitrogen Profile of PBII Nitrided Titanium Alloy

Valérie Parry [1], Eric Le Bourhis [2,*], Luc Pichon [2] and Michel Drouet [2]

Figure S1. Reduced elastic modulus profiles for 3 treatments at 600 °C, 700 °C and 800 °C.

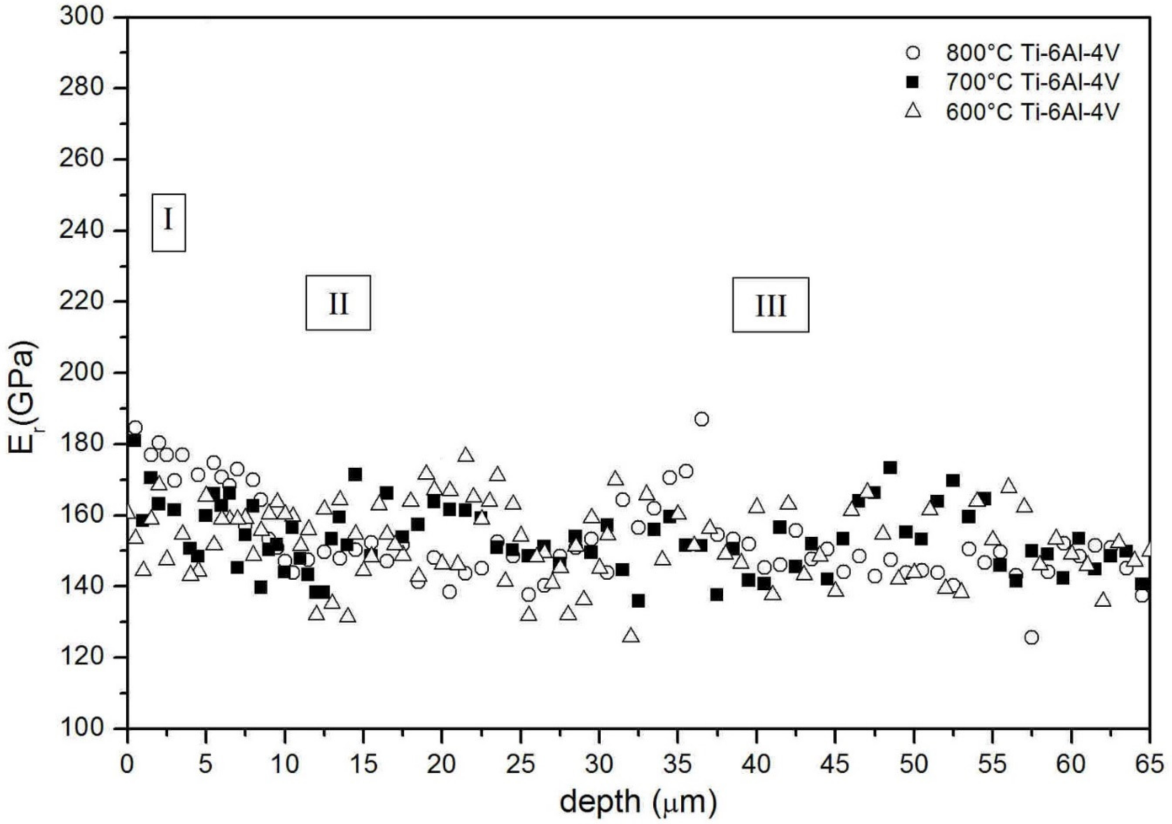

Supplement: Supplementary file 1 [file materials-15-09028-s001.zip › materials-2054762-supplementary.pdf]
